# Role of chiral quantum Hall edge states in nuclear spin polarization

**DOI:** 10.1038/ncomms15084

**Published:** 2017-04-20

**Authors:** Kaifeng Yang, Katsumi Nagase, Yoshiro Hirayama, Tetsuya D. Mishima, Michael B. Santos, Hongwu Liu

**Affiliations:** 1 State Key Lab of Superhard Materials, Institute of Atomic and Molecular Physics, Jilin University, Changchun 130012, China; 2 Department of Physics, Tohoku University, Sendai, Miyagi 980-8578, Japan; 3 Homer L. Dodge Department of Physics and Astronomy, University of Oklahoma, 440 West Brooks, Norman, Oklahoma 73019-2061, USA

## Abstract

Resistively detected NMR (RDNMR) based on dynamic nuclear polarization (DNP) in a quantum Hall ferromagnet (QHF) is a highly sensitive method for the discovery of fascinating quantum Hall phases; however, the mechanism of this DNP and, in particular, the role of quantum Hall edge states in it are unclear. Here we demonstrate the important but previously unrecognized effect of chiral edge modes on the nuclear spin polarization. A side-by-side comparison of the RDNMR signals from Hall bar and Corbino disk configurations allows us to distinguish the contributions of bulk and edge states to DNP in QHF. The unidirectional current flow along chiral edge states makes the polarization robust to thermal fluctuations at high temperatures and makes it possible to observe a reciprocity principle of the RDNMR response. These findings help us better understand complex NMR responses in QHF, which has important implications for the development of RDNMR techniques.

## Abstract (web summary)

Quantum Hall phases in two-dimensional systems have chiral edges, along which electrons propagate in one direction without backscattering. Here, the authors use nuclear magnetic resonance to demonstrate how chiral modes establish dynamical nuclear polarization in a quantum Hall ferromagnet.

Resistively detected NMR (RDNMR)^1^ developed in a quantum Hall ferromagnet (QHF)^2^,^3^,^4^ of GaAs two-dimensional electron gases (2DEGs) at filling factor *ν*=2/3 (corresponding to a composite-fermion filling factor ν*=2)^5^,^6^ has been widely used to discover exotic 2D electronic states^7^,^8^,^9^,^10^ and to coherently control the nuclear spins in 2DEGs^11^. The RDNMR technique depends on the current-induced dynamic nuclear polarization (DNP) that is expected to occur by transferring spin polarization from electrons to nuclei via the electron–nuclear hyperfine interaction at a domain wall (DW) separating two energetically degenerate domains^12^. This polarization process is in contrast to DNP by electron spin resonance^1^ or optical^13^,^14^,^15^ pumping of intra- or inter-band transitions to generate nonequilibrium electron spin polarizations, which then polarize the nuclei via the hyperfine interaction during subsequent relaxation to equilibrium. However, the detailed mechanism is still poorly understood. In particular, the above-mentioned studies of the *ν*=2/3 QHF are performed on the Hall bar where contributions from both bulk and edge states to DNP^16^,^17^,^18^,^19^ coexist and also the edge physics at *ν*=2/3 remains unclear^20^, which may complicate its interpretation.

The *ν*=2/3 QHF is classified as an easy-axis ferromagnet according to its magnetic anisotropy energy^4^. Such ferromagnetic ground states have also been formed in integer QH regimes of various 2DEGs^21^,^22^,^23^,^24^,^25^. It is known that QH edge states at integer *ν* correspond to bulk Landau levels (LLs) below the Fermi energy^26^, which are chiral in the sense that they propagate in only one direction on a given edge of a Hall bar—the right-moving state on the top edge and the left-moving one on the bottom edge (or vice versa, depending on the orientation of magnetic field). The chiral edge states are immune to backscattering and localization provided there is no interedge scattering^27^, which accounts for a non-dissipative (quantized) transport in the QH effect^28^.

Here we focus on the edge state in the dissipative transport of QHF where its chiral character has received little attention and show how chiral modes establish DNP. We present comparative RDNMR measurements of the simplest easy-axis QHF at *ν*=2 of InSb 2DEGs^29^ patterned into Hall bar and Corbino disk configurations. The absence of edge states in the Corbino disk allows us to investigate DNP in bulk^16^, which provides a basis for discussion of DNP via edge states of the Hall bar. This side-by-side comparison experiment reveals a reciprocity principle of the NMR response in the *ν*=2 QHF at temperatures where the bulk contribution to DNP vanishes but the intraedge-scattering-induced DNP still operates, highlighting the important role of chiral edge states on DNP. Our results clearly show that the chiral edge state has direct effects on the nuclear spin polarization besides its known effects on the electron transport in QH systems.

## Results

### RDNMR measurements of the Corbino disk

**Figure 1:**
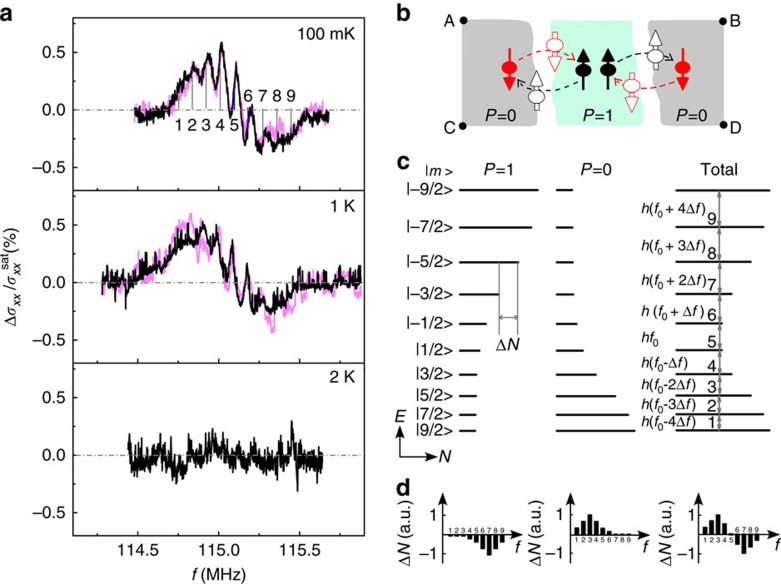
Temperature dependence of direct current (DC) RDNMR spectra of ^115^In in a Corbino disk. (**a**) 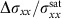 versus *f* as a function of temperature at *B*=12.3 T with current *I*=0.6 μA (black curve) and *I*=−0.6 μA (pink cruve). The dashed–dotted line represents the zero level. Quadrupole resonances are indicated by vertical solid lines with numbers 1–9. (**b**) Schematic domain structures of quantum Hall ferromagnet (QHF). The grey and green areas denote the spin-unpolarized (spin polarization *P*=0, spin-up (black solid arrow) and spin-down (red solid arrow) electrons in the two Zeeman levels of the *n*=0 Landau level (LL), see inset of Supplementary Fig. 4) and spin-polarized (*P*=1, spin-up electrons in both *n*=0 and *n*=1 LLs) domains, respectively, and a domain wall (DW) occurs in between. For clarity, spin-up electrons in the *n*=0 LL are not shown in the graph. The electron-spin flip (say from spin-down to spin-up, red dashed arrows) flops one nuclear spin from spin-up (black hollow arrow) to spin-down (red hollow arrow) at DW boundaries. Note that the arrow length is not scaled with the magnetic moment of each particle. (**c**) A possible population distribution (energy *E* versus population *N*) of ^115^In with 10 nuclear spin states |*m*> near the *P*=0 and *P*=1 domains and the total population distribution by assigning the same weight from these two domains to the RDNMR response. The presence of electric quadrupole coupling accounts for a difference in the splitting between these levels (where *f*_0_ and Δ*f* are the Zeeman and quadrupole frequencies, respectively, and *h* is Planck's constant). (**d**) The corresponding population difference between adjacent levels (denoted by numbers 1–9), Δ*N*=*N*_|*m*>_−*N*_|*m*-1>_, as a function of *f*, where the frequency interval is equally spaced by Δ*f* and the largest Δ*N* is taken as unity. The total Δ*N*−*f* dependence is proposed to be responsible for quadrupole resonances in data that are equally spaced by Δ*f*∼85 kHz.

The magnetoresistance and RDNMR measurements of InSb 2DEGs were performed in a dilution refrigerator (see Methods section). A comparative RDNMR study was carried out on the *ν*=2 QHF of the two configurations that was a highly sensitive region for the detection of DNP (see Methods section), focusing on the dependence of RDNMR signals on the type (alternating current (AC) or direct current (DC)) of electric current, the direction of current flow, the orientation of magnetic field and the effect of temperature. We first present the results obtained from the Corbino disk. Figure 1 shows that the DC RDNMR signal of ^115^In has a dispersive line shape (DLS) with quadrupole splittings at low temperatures (*T*) and disappears at 2 K. Note that the signals at *T* ≤1 K are nearly independent of temperature due to the current-induced heating^30^. Figure 1b depicts the domain structures of QHF, where the spin-polarized (spin polarization *P*=1) and spin-unpolarized (*P*=0) domains are separated by DW (order of the magnetic length *l*_B_ in width^31^). Charge transport across the DW accompanying electron spin flip between two energetically degenerate domains is responsible for an emerging conductivity spike that characterizes the QHF (Supplementary Note 1). Although compared to the hyperfine interaction the spin-orbit coupling (large in InSb) is much more efficient to flip electron spins, the role played by nuclear spins is still evident from the observed RDNMR signal that is generally accepted to be caused by DNP at DW boundaries via electron–nuclear flip-flop^12^. The interplay between the hyperfine and spin-orbit coupling in the RDNMR sensitivity deserves future study. The electric field gradient at nuclear positions induced by strain^19^ in the InSb QW accounts for the quadrupole coupling to ten nuclear energy levels of ^115^In with nuclear spin *I*_*N*_=9/2 (ref. 32). Single-photon transitions among these levels are expected to result in nine quadrupole resonances with equal frequency intervals. Because the conductivity change Δ*σ*_*xx*_ (see Methods section) is determined by a population (*N*) of each nuclear energy level that depends on the spin configuration of electrons coupled to nuclei^33^, we propose that the nuclear population profiles near the *P*=1 and *P*=0 domains are different (Fig. 1c). The population difference Δ*N* (Fig. 1c,d) will increase (decrease) the Zeeman splitting *E*_Z_ of electron spins in the *P*=1 (*P*=0) domain via the Overhauser effect, which is equivalent to increasing (decreasing) a parallel field but keeping the perpendicular component *B*_perp_ constant in a tilted-magnetic-field measurement because the Overhauser effect has no influence on electron orbital motion determined by *B*_perp_. This results in a leftward (rightward) shift of the conductive spike after the DNP^29^ and thus a dip (peak) in the RDNMR signal. The DLS in data suggests that the polarized nuclear spins near the *P*=1 and *P*=0 domains separated by a domain size (several hundreds of nm^34^) give the same weight to the RDNMR response as all possible electron trajectories across the DW between the two sides (A-C and B-D) are involved (called bulk mode). This interpretation is further supported by the fact that the peak-to-dip pattern is reversed as the signal is taken from the other side of the spike (data not shown). From the above discussion, it follows that the DC RDNMR signal is independent of the direction of current flow (Fig. 1a) and the orientation of magnetic field (Supplementary Fig. 1). In addition, the DC signal is found to be cool-down independent (Supplementary Fig. 2).

In contrast to the DC measurement where the polarized nuclei with opposite spins are well separated, the current flow with opposite directions in the AC measurement produces the two species at both sides of one DW that are gradually distributed over a narrow width^12^. In this case, the nuclear spin polarization is negligible on average that is responsible for the absence of RDNMR signals (Supplementary Fig. 3). The role of AC current is also played by high temperatures in the DC measurement, where thermal fluctuations make the direction of electron transfer between neighbouring domains^35^ opposite to that driven by the DC current and thus suppress the RDNMR signal (Fig. 1a).

### RDNMR measurements of the Hall bar

**Figure 2:**
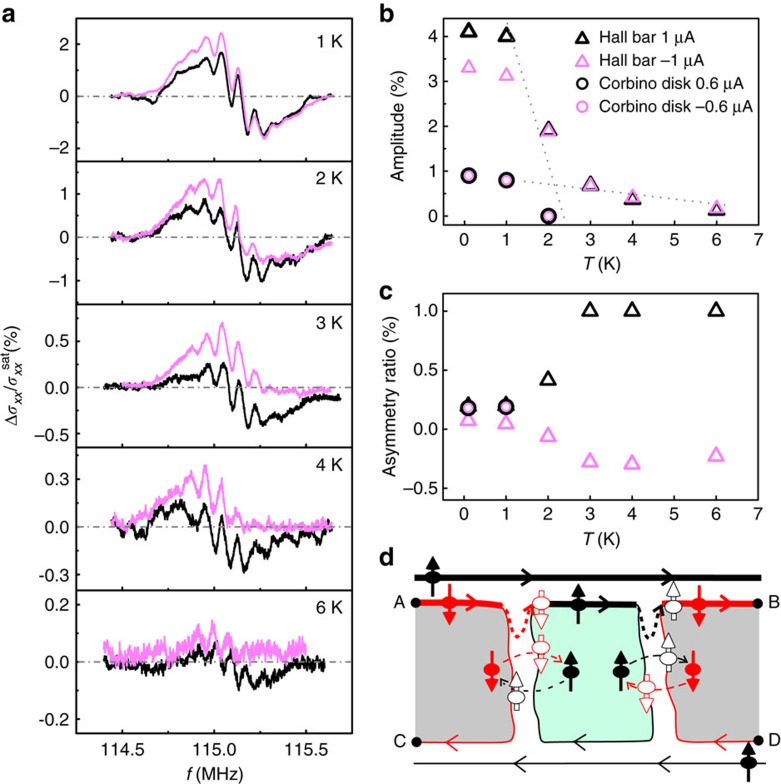
Temperature dependence of DC RDNMR spectra of ^115^In in a Hall bar. (**a**) 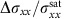 versus *f* as a function of temperature at *B*=12.3 T with *I*=1 μA (black curve) and *I*=−1 μA (pink curve). The dashed–dotted line represents the zero level. (**b**,**c**) Temperature dependence of the amplitude (peak-to-dip height) of 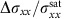 and the asymmetry ratio of the height difference between peak and dip to the amplitude of 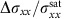 obtained from the data of (**a**) and Fig. 1. The dotted lines in (**b**) are guides for the eye. (**d**) Schematic domain structures of QHF. The top and bottom lines denote the edge state corresponding to the pseudospin-up ((*n*, *σ*)=(0,↑)) LL and the solid line surrounding domains denotes the one corresponding to either the pseudospin-down ((*n*, *σ*)=(0,↓)) or pseudospin-up ((*n*, *σ*)=(1,↑)) LL. Line thickness represents the relative intensity of edge current. Electron–nuclear flip-flop via the bulk (edge) state is indicated by thin (thick) dashed arrows.

The results obtained from the Corbino disk provide a good reference for the RDNMR study of the Hall bar where both bulk and edge states coexist. The temperature-dependent DC RDNMR spectrum of the Hall bar is shown in Fig. 2a, which is found to be significantly different from that of the Corbino disk. In particular, the signals are present at high temperatures up to 6 K with changes in line shape. For ease of comparison, we summarize the results of a detailed analysis of the signals of both configurations in Fig. 2b,c. It is clear that the two configurations have a DLS with relatively symmetric peak and dip heights for both directions of current flow at *T* ≤1 K despite the large difference in signal amplitude. The signal amplitude of the Corbino disk goes directly to zero as the temperature is raised to 2 K, while that of the Hall bar decreases rapidly with increasing temperature first and then shows a gradual decrease from 3 to 6 K. Furthermore, the signal asymmetry ratio in this temperature range is large and almost constant, with the sign depending on the direction of current flow. These findings lead us to conclude that DNP is dominated by the bulk mode at low temperatures and the presence of edge states in the Hall bar is responsible for the observed differences (see below). Figure 2d depicts the domain structures of QHF in the Hall bar, where the edge states become part of an array of domains. It is seen that the two edges of the sample are connected by channels along domain boundaries, which is supported by optically detected magnetic resonance imaging of the *ν*=2/3 QHF of GaAs 2DEGs^20^. The edge transport will affect the bulk mode of the Hall bar as follows: the chiral nature of edge states determines that electrons feeding into the sample from one side (for example, point A) either go back to the same side (point C) by travelling along the DW without spin flip or reach the other side (point B) by passing across the DW with spin flip^36^. Although the former process does not contribute to DNP directly, it tends to reduce the number of electrons passing across the DW that not only results in the spike with high resistance (or *σ*_*xx*_, Supplementary Fig. 4) but also improves the RDNMR sensitivity as indicated by a large signal amplitude in Fig. 2b. Furthermore, the edge transport is also responsible for the dependence of signal amplitude of the Hall bar on the direction of current flow (Fig. 2b), the orientation of magnetic field (Supplementary Fig. 1) and different cool-downs (Supplementary Fig. 2), as discussed in Supplementary Note 2.

### Reciprocity principle of the RDNMR signal

**Figure 3:**
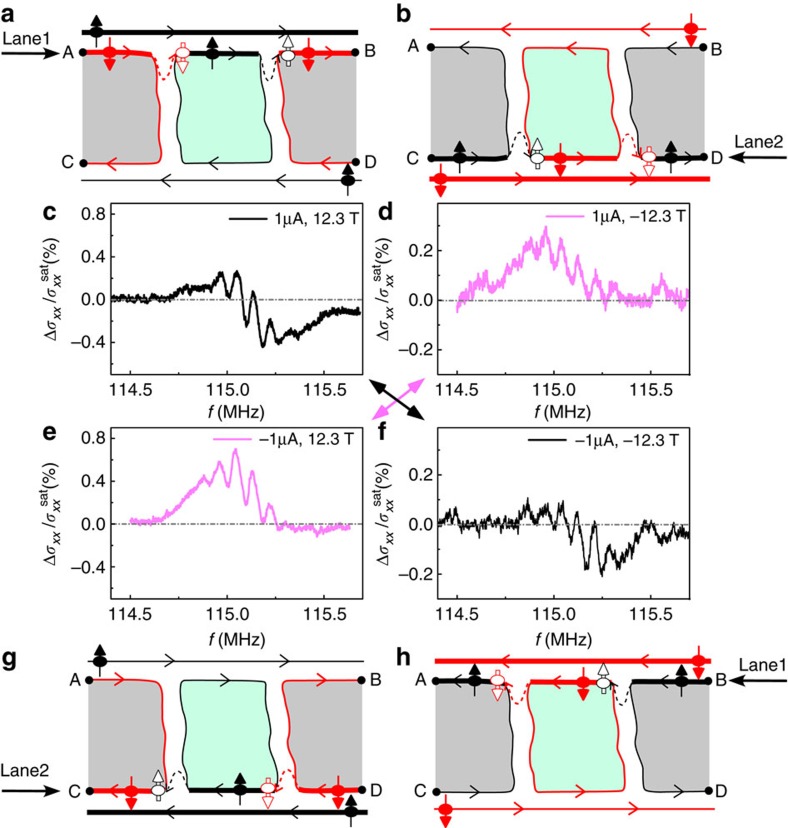
Reciprocity principle of the RDNMR response in a Hall bar. DC RDNMR spectra of ^115^In measured at *T*=3 K, *B*=±12.3 T and *I*=±1 μA in panels (**c**–**f**) together with the corresponding schematic domain structures of QHF in panels (**a**,**b**,**g**,**h**) show that the RDNMR line shape depends on which edge current path mainly contributes for DNP: one between points A and B (called lane 1) and the other between points C and D (lane 2). This reciprocal RDNMR response is obtained by requiring both current flow and magnetic field to be reversed. The dashed–dotted line represents the zero level.

A distinct change in the temperature dependence of signal amplitude and asymmetry ratio of the Hall bar suggests that the mechanism of DNP may change from the bulk mode to the edge mode (that is, DNP due to electron–nuclear flip-flop via edge states with opposite spins as indicated by thick dashed arrows in Fig. 2d, also called intraedge-scattering-induced DNP). This is further supported by the observation of a reciprocity principle of the RDNMR response in Fig. 3. For the edge-state picture of the QH effect, the chiral nature (that is, the one-way electron motion) makes a difference in current between the top and bottom edge states when a driving current is induced, as denoted by line thickness in Fig. 3a,b,g,h. This difference makes the DNP mainly occur along either of the two spatially separated paths of lane 1 and lane 2, resulting in different signal line shapes in Fig. 3c–f. The signal line shape is found to be unchanged provided the lane mainly responsible for DNP is kept the same by simultaneously reversing both current flow and magnetic field (Fig. 3a,b,g,h), while the direction of electron motion along the same lane only changes the signal amplitude. Note that, because the reversal of current difference between the two lanes is compatible with that of a Hall voltage, we can also say that the reciprocity principle in Δ*σ*_*xx*_ similar to the Hall voltage occurs in the edge-dominated region. It follows from the signal line shape and the above discussion that the polarized nuclear spins near the *P*=1 (*P*=0) domain give more weight to the RDNMR response as electrons move along lane 1 (lane 2). The chiral nature also ensures that electron motion along the edge state is unidirectional and thus robust to thermal fluctuations, accounting for the presence of RDNMR signals at high temperatures. It is worth noting that the signal amplitude determined by the thermally robust edge mode is small and less sensitive to temperature (Fig. 2b), indicating that the edge mode is overwhelmed by the bulk mode at low temperatures. Therefore, we conclude that DNP is dominated by the bulk mode at low temperatures but by the edge mode at high temperatures when the bulk mode is completely suppressed. This edge mode is also present in the AC RDNMR measurement of the Hall bar where DNP in bulk is suppressed (Supplementary Note 2).

## Discussion

The observed reciprocity principle of the NMR response also helps us better understand DLS of the *ν*=2 QHF. We infer from Fig. 3 that electron trajectories in the bulk mode can be regarded as convergence of the two lanes in the edge mode zig-zagging throughout the 2D plane with the same transmission probability and current flow direction but without the chiral nature, resulting in DLS. As discussed above, the DNP-induced decrease (increase) of the electronic Zeeman energy near the *P*=0 (*P*=1) domain results in the peak (dip) feature and the difference between peak and dip heights is determined by the weight of these two domains given to the NMR response. Furthermore, as compared with the Δ*N*−*f* dependence in Fig. 1d, the emergence of the fifth resonance line in data is suggestive of the Knight shift (*K*_s_) of the RDNMR response near the *P*=1 domain relative to that near the *P*=0 domain. The frequency spacing between peak and dip of this resonance gives *K*_s_∼35 kHz. Note that, because the quadrupole splitting Δ*f*∼85 kHz is much larger than *K*_s_ in our case, the frequency spacing between peak and dip of the DLS as a whole is determined by the quadrupole splittings (∼5Δ*f* ) rather than by *K*_s_. With this understanding, we now proceed to investigate the possible role of domain structures in the *ν*=1 DLS of the GaAs 2DEG whose origin is a long-standing mystery^37^,^38^,^39^,^40^,^41^,^42^,^43^. The most recent research^44^,^45^ confirms that the peak and dip signals are attributed to the coupling of nuclei to spin-unpolarized and spin-polarized 2DEGs, respectively, and the frequency spacing between them is determined by *K*_s_ (this determination is consistent with ours made at the fifth resonance line). To explain these findings, it is further proposed that the 2DEG near *ν*=1 could spontaneously break symmetry to form domains with polarized and unpolarized regions. However, we have to note that, in contrast to the *ν*=2 QHF where the current-induced DNP induces opposite changes in the electronic Zeeman energy of different domains that is responsible for DLS, the assigned thermal nuclear polarization near *ν*=1 (ref. 37) can only decrease the electronic Zeeman energy and result in a dip. Thus the NMR-induced heating of the 2DEG together with the Zeeman effect has been considered^40^ but ruled out by recent experiments^44^,^45^,^46^. One possibility to account for the *ν*=1 DLS using the domain scenario is that the current applied to *ν*=1 with domain structures in the literature might be large enough to induce DNP that exceeds thermal nuclear polarization. Further studies following our work are required to examine this possibility.

Finally, we note that our understanding of the role of chiral edge states in DNP and complex NMR responses in the simplest easy-axis QHF at *ν*=2 may shed light on the study of DNP in the *ν*=2/3 (or ν*=2) QHF. In addition, the RDNMR spectra at *ν*=2/3 have recently been used to determine the topology of various QH phases (stripe, bubble, Wigner and Skyrme crystals)^9^,^10^,^47^,^48^, where the Knight shift of all nuclei that have considerable overlap with the electron wave function is summed to calculate the signal line shape. We emphasize that attention should be paid to the spatial distribution of DNP varying with sample configurations and experimental conditions as discussed in our work, which may affect the profile of sub-band wavefunctions at filling factors used for the signal readout and thus the NMR line shape.

## Methods

### Sample preparation and characterization

The 2DEG in a 20-nm-wide InSb quantum well grown on GaAs (001) substrates^49^ (Supplementary Fig. 5c) was patterned into Corbino disk and Hall bar configurations (Supplementary Fig. 5a,b) simultaneously on one chip that was subjected to the same measurement procedures for our comparison purpose. Indium was used for Ohmic contacts in both samples. The Corbino disk was defined by two circular Ohmic contacts (source S and drain D) with radii of *r*_1_=95 μm and *r*_2_=195 μm, respectively, and the Hall bar had a length of *L*=100 μm and a width of *W*=30 μm. The following 2DEG parameters were measured on the Hall bar at *T*=100 mK in a dilution refrigerator equipped with *in situ* rotator stage using a standard AC lock-in technique at 13.7 Hz. Determination of the tilt angle *θ* between the sample normal and *B* (Supplementary Fig. 4, inset) was made by measuring the slope of low-field Hall resistance. The electron mobility (*μ*) and density (*n*_s_) were obtained from the Hall measurement at *θ*=0° and found to be cool-down dependent (for example, *μ*=20 (20.6) m^2^/Vs and *n*_s_=2.66 (2.7) × 10^15^ m^−2^ for the first (second) cool-down). The effective mass m*∼0.016 in units of the free-electron mass *m*_e_ was determined by analysing the temperature-dependent amplitude of low-field Shubnikov-de Haas oscillations^50^. The coincidence technique was used to measure the product of m*g* at the LL intersection and thus the effective *g*-factor g* that was shown to be linear with spin polarization of each LL intersection^50^. The QHF spike studied here was formed at the *ν*=2 LL intersection with *θ*=64.1° (Supplementary Fig. 4), where g*∼55 was obtained.

### RDNMR measurement

A low-noise preamplifier (Stanford Research Systems, Model SR560) and a standard AC lock-in technique at 13.7 Hz were used for the DC and AC RDNMR measurements, respectively, at temperatures from 100 mK to 6 K. The RDNMR measurements were performed on the *ν*=2 QHF formed at the energy gap *ɛ*=0 (Supplementary Fig. 4, inset). The details of the RDNMR measurement are as follows: a large current is applied to polarize the nuclei around the *ν*=2 spike, as indicated by an exponential increase in *σ*_*xx*_ on a timescale of hundreds of seconds (Supplementary Fig. 6a). After *σ*_*xx*_ becomes saturated (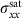), a continuous-wave radio-frequency field (∼μT) at a power of 0 dBm generated by a single turn coil surrounding the sample is applied to irradiate the 2DEG. The change in *σ*_*xx*_ with respect to 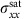 during frequency (*f*) sweep through the resonance condition of *f*_NMR_=*γB* (*γ*, the gyromagnetic ratio of ^115^In) defines Δ*σ*_*xx*_, which describes the depolarization of nuclei. A slow sweep rate (12 kHz/min) is used in order that *σ*_*xx*_ at each frequency point approaches the equilibrium value. The *f* dependence of 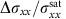 represents the RDNMR spectrum. Note that an increase in *n*_s_ for the second cool-down results in a shift of the *ν*=2 spike towards higher magnetic fields and thus a difference in the field strength (12 and 12.3 T for the first and second cool-downs, respectively) at which the RDNMR measurement is performed.

### Detection sensitivity

As the gap *ɛ* is made to approach zero by adjusting *θ* (Supplementary Fig. 4, inset), *ɛ* and thus the *ɛ*=0 position are strongly influenced by the hyperfine contribution to the electronic Zeeman splitting, *Δ*_HF_=Σ_*j*_*A*^(*j*)^ <*I*^(*j*)^> (where *A*^(*j*)^ and <*I*^(*j*)^> are the hyperfine interaction constant and nuclear spin polarization of different nuclei *j*, respectively^1^). This results in a shift of the QHF spike and allows detection of the RDNMR signal, which is similar to the RDNMR measurement of the *ν*=2/3 QHF in the GaAs 2DEG^7^. We calculate that the two systems have a comparable Δ_HF_ (446 μeV in InSb and 140 μeV in GaAs) if all nuclei are fully polarized (that is, <*I*^(*j*)^> is equal to the nuclear spin *I*_N_ of each nuclear isotope). A relatively large Δ_HF_ in InSb is mainly due to the large *I*_N_. It is estimated that the degree of nuclear polarization (*P*_N_) in the *ν*=2 QHF of the InSb 2DEG is about 10% (see below), which is comparable to that in the *ν*=2/3 QHF of the GaAs 2DEG^51^. This degree of polarization results in 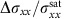 on the order of a few percent. From the above discussion, it follows that QHF is a highly sensitive region for the RDNMR measurement. Here we note that the effective nuclear field *B*_N_=Δ_HF_/(g*μ_*B*_) in InSb is extremely small due to large g*: an absolute value |*B*_N_| in InSb with g*∼−55 is only about 0.14 T if <*I*^(*j*)^>=*I*_N_ while that in GaAs with g*∼−0.44 is ∼5.3 T. In our study, |*B*_N_|∼0.014 T is calculated by *B*_N_=*B*_perp_/cos(*θ*+Δ*θ*)−*B*_perp_/cos*θ*, where Δ*θ* is an equivalent change in angle due to DNP that is deduced from 
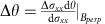
 with 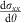 obtained from the angle dependence of the spike position^29^. Therefore, we have *P*_N_=0.014 T/0.14 T=10%.

### Data availability

The data that support the findings of this study are available from the corresponding author upon request.

## Additional information

**How to cite this article:** Yang, K. *et al*. Role of chiral quantum Hall edge states in nuclear spin polarization. *Nat. Commun.* **8,** 15084 doi: 10.1038/ncomms15084 (2017).

**Publisher's note:** Springer Nature remains neutral with regard to jurisdictional claims in published maps and institutional affiliations.

## Supplementary Material

Supplementary Information Supplementary Figures, Supplementary Notes and Supplementary References
