# Supplementary material for: Role of chiral quantum Hall edge states in nuclear spin polarization
Source: Nat Commun. 2017 Apr 20;8:15084. doi: 10.1038/ncomms15084 (PMC5411482; doi:10.1038/ncomms15084)
Supplement: Supplementary Information — Supplementary Figures, Supplementary Notes and Supplementary References [file ncomms15084-s1.pdf]

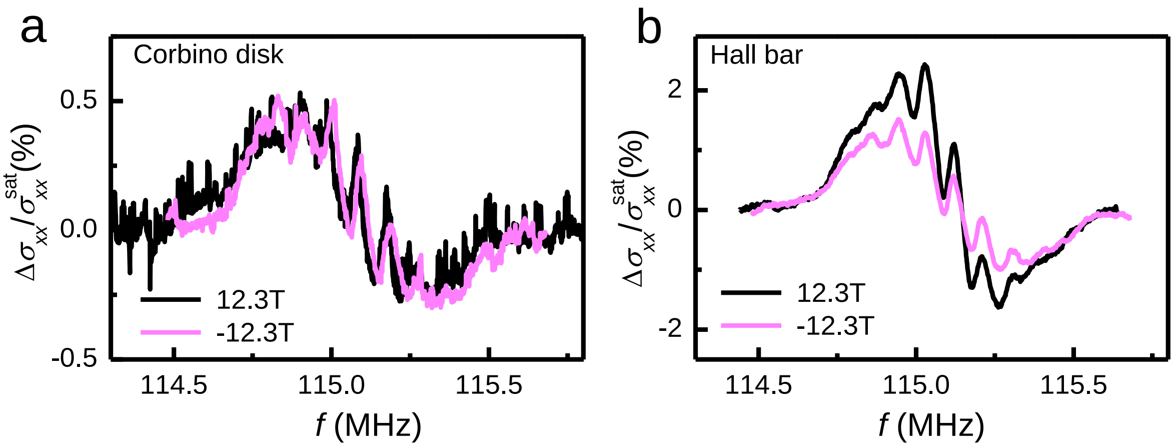

Supplementary Figure 1: Direct current (DC) RDNMR signals at different *B* orientations. $\Delta\sigma_{xx} / \sigma_{xx}^{sat}$ versus *f* for both Corbino disk (**a**) and Hall bar (**b**) at $B = \pm 12.3$ T and $T = 100$ mK.

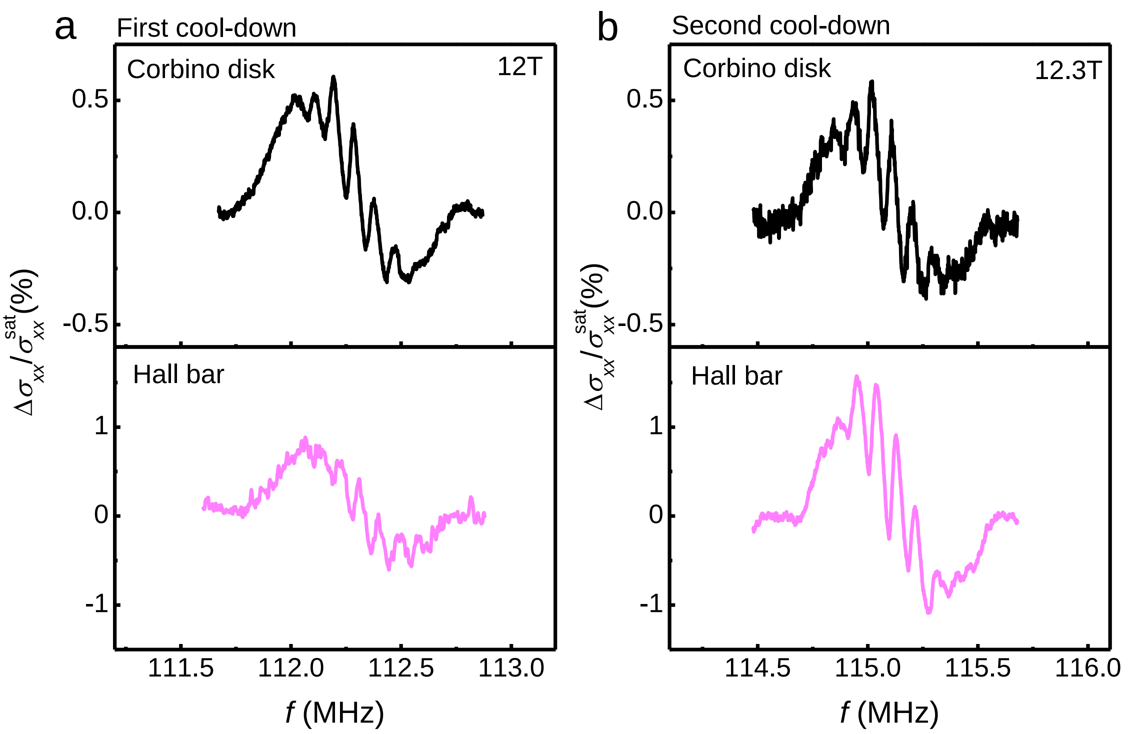

Supplementary Figure 2: DC RDNMR signals for different cool-downs. $\Delta\sigma_{xx} / \sigma_{xx}^{\text{sat}}$ versus f for both Corbino disk and Hall bar measured after the first (**a**) and second (**b**) cool-downs to T = 100 mK. Note that the amplitude scale is different for the two samples.

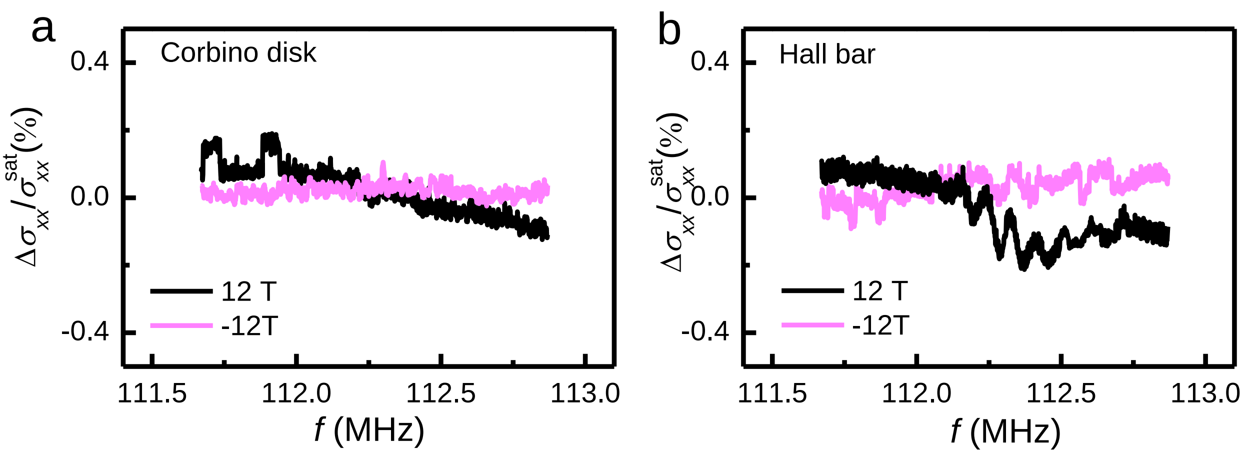

Supplementary Figure 3: Alternating current (AC) RDNMR signals at different *B* orientations. $\Delta\sigma_{xx} / \sigma_{xx}^{\mathrm{sat}}$ versus f for both Corbino disk (**a**) and Hall bar (**b**) at $B = \pm 12$ T and $T = 100$ mK.

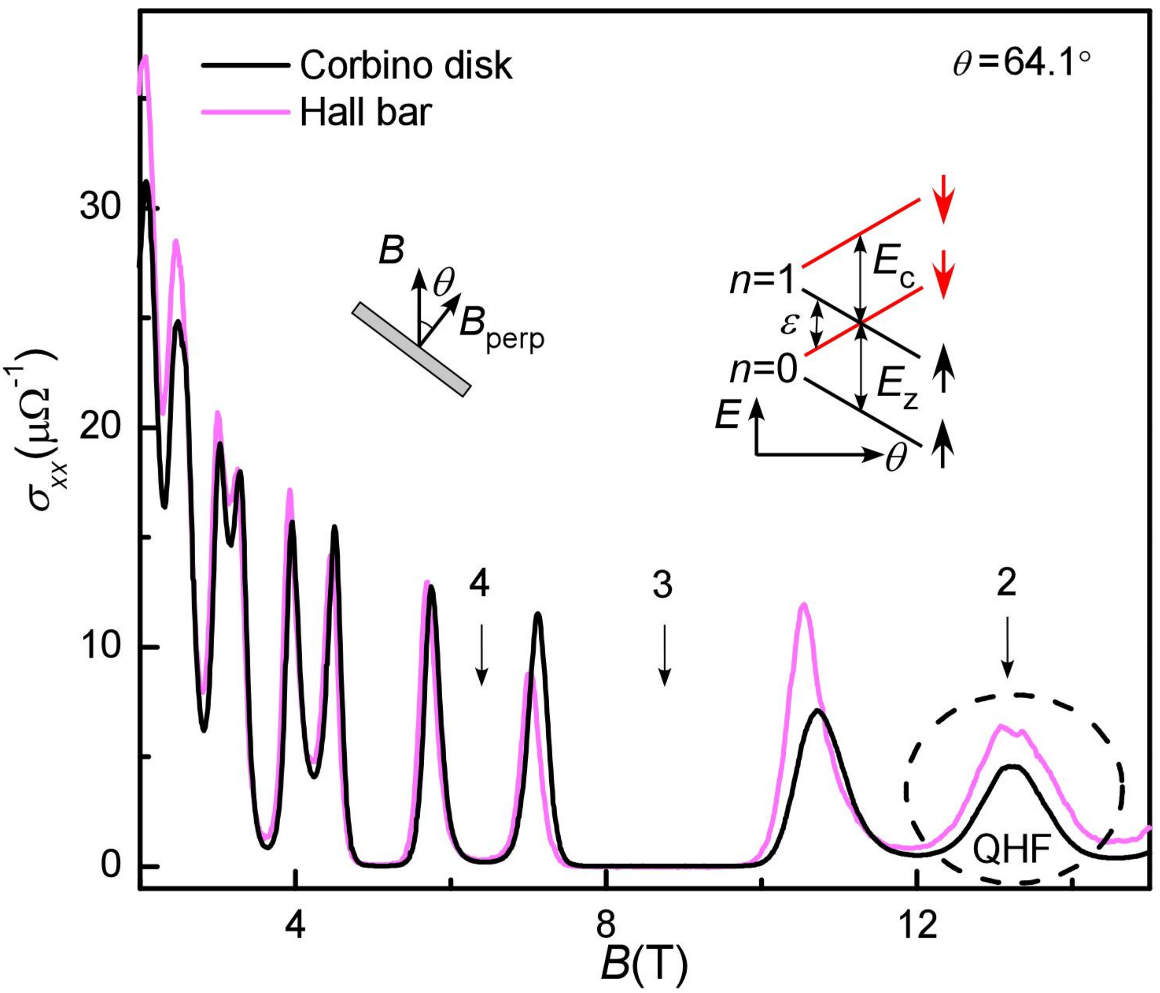

Supplementary Figure 4: Quantum Hall ferromagnet (QHF). σ_{xx} versus B for both Corbino disk and Hall bar with a tilt angle of $\theta = 64.1°$ (θ is the angle between B_{perp} along the sample normal and B shown in the left inset) measured after the first cool-down to $T = 100$ mK. The conductivity spike at $\nu = 2$ marked by circles is a signal of QHF that is formed at the LL intersection with the energy gap $\varepsilon = 0$ when the Zeeman splitting of $E_z = g^* \mu_B B = g^* \mu_B B_{\text{perp}} / \cos\theta$ (where μ_B is the Bohr magneton) and the cyclotron splitting of $E_{\text{c}} = \hbar e B_{\text{perp}} / m^*$ (where $\hbar$ is the reduced Planck's constant and e is the electron charge) are made equal by adjusting θ (right inset).

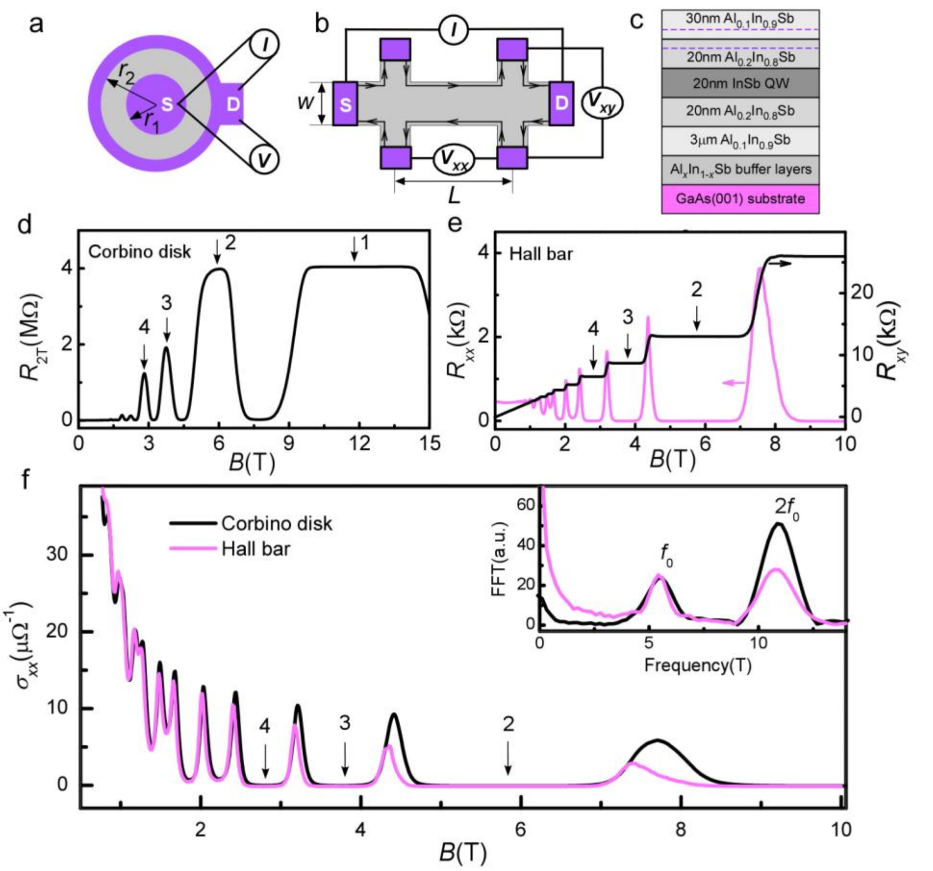

Supplementary Figure 5: Quantum Hall effect. Corbino disk (**a**) and Hall bar (**b**) configurations fabricated on the two-dimensional electron gas (2DEG) in an InSb quantum well (**c**). The Corbino disk is defined by two circular Ohmic contacts (source S and drain D) with radii of r_1 = 95 μm and r_2 = 195 μm, respectively. The S-D current (I) and output voltage (V) give a two-terminal resistance R_{2T}. The Hall bar has a length of L = 100 μm and a width of W = 30 μm. The S-D current I and output voltage V_{xx} (V_{xy}) give longitudinal resistances R_{xx} (Hall resistances R_{xy}). The trajectory of electrons carried by edge states along the sample boundary whose direction is determined by the orientation of B is shown in **b**. **d**, R_{2T} versus B. The number indicates the Landau-level (LL) filling factor ν. **e**, R_{xx} and R_{xy} versus B. **f**, σ_{xx} versus B for both Corbino disk and Hall bar calculated by $\sigma_{xx} = \ln(r_2/r_1)/(2\pi R_{2T})$ and $\sigma_{xx} = \rho_{xx}/(\rho_{xx}^2 + \rho_{xy}^2)$ $(\rho_{xx} = R_{xx}W/L; \rho_{xy} = R_{xy})$, respectively. Inset shows the fast Fourier transform (FFT) spectrum taken from the low-field SdH oscillations of the two configurations, from which $n_s = 2ef_0/h = 2.66 \times 10^{15}\,\mathrm{m}^{-2}$ is obtained. All measurements were performed after the first cool-down to T = 100 mK.

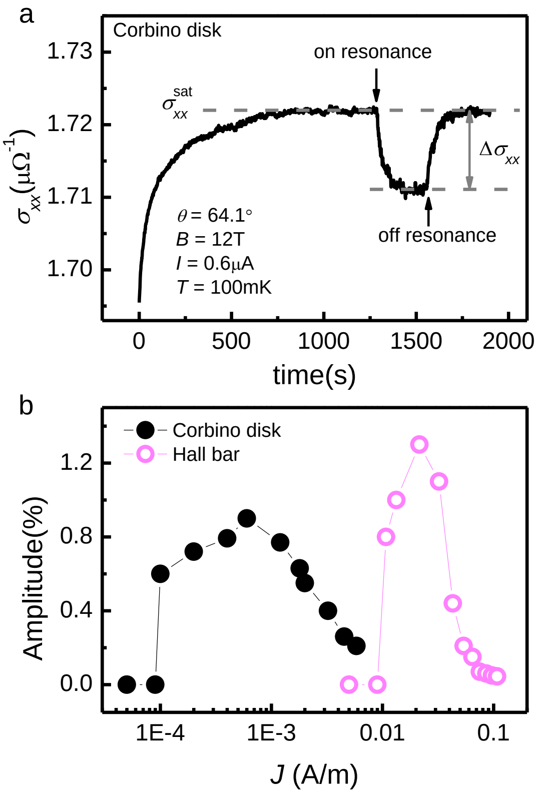

Supplementary Figure 6: Current dependence of RDNMR signal amplitude. a, Time dependence of $\Delta\sigma_{xx}$ in the Corbino disk. The "on resonance" ("off resonance") condition corresponds to the radio frequency (f) matching (mismatching) the gyromagnetic ratio of ^{115}In. **b**, Amplitude (peak to dip) of $\Delta\sigma_{xx} / \sigma_{xx}^{sat}$ versus the DC density (J) for both Corbino disk ($J = I/2\pi r_1$) and Hall bar ($J = I/W$) at B = 12 T and T = 100 mK. Because J has a radial dependence in the Corbino disk, the calculation at inner circular contacts gives an upper bound. Note that a decrease in amplitude with increasing J for both samples is due to the nuclear depolarization caused by the current-induced heating.

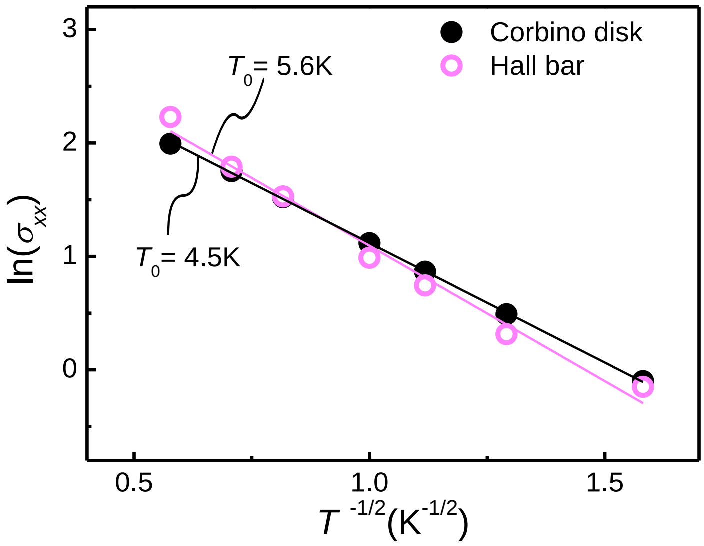

Supplementary Figure 7: Variable-range hopping transport. Temperature dependence of σ_{xx} at B = 12 T around which the RDNMR measurement was performed (Supplementary Fig.4). The lines are the fits to the variable-range hopping formula $\sigma_{xx} \propto \exp\left(-\sqrt{T_0 / T}\right)$ (T_0, the characteristic temperature).

Supplementary Note1: Magnetotransport in the quantum Hall and quantum Hall ferromagnet regimes

We examined the quantum Hall effect and magnetotransport properties of both Corbino disk and Hall bar using the alternating current (AC) measurement in a magnetic field (B) perpendicular to the sample substrate. It is shown in Supplementary Fig. 5d,e that the Corbino disk has high resistance $R_{2T} \sim M\Omega$ near an integer filling factor ν where the Hall bar has zero longitudinal resistance R_{xx} with the Hall plateau. Electrons in the Corbino disk are trapped in localized bulk states at integer ν that makes it electrically insulating, while those in the Hall bar are carried by either edge channel or Hall current path in bulk that accounts for a non-dissipative transport. Although the two samples have different magnetoresistance properties, they have a similarity in diagonal conductivity σ_{xx} (Supplementary Fig. 5f) that is most directly related to the Landau-level (LL) nature and characterizes the bulk dissipation near the Fermi energy[1]. The σ_{xx}-B dependence at low fields is found to be consistent with each other, suggesting the same electron mobility and density in both samples. Note that an asymmetric line shape of the $\sigma_{xx}(B)$ peak for the Hall bar at high fields is caused by the difference in coupling efficiency between the edge and bulk states as LLs are raised above the Fermi level with increasing B[2]. This does not occur for the Corbino disk without edge states.

We constructed and characterized the quantum Hall ferromagnet (QHF) of both Corbino disk and Hall bar using the AC measurement in tilted magnetic fields. The QHF is formed at $\nu = 2$ where the pseudospin-down [$(n, \sigma) = (0,\downarrow)$] and pseudospin-up [$(n, \sigma) = (1,\uparrow)$] LLs ($n$ and σ are the orbital and spin indices, respectively) are brought into degeneracy (i.e., the single-particle energy gap ε is zero) by modifying the tilt angle θ to balance the Zeeman and cyclotron splitting (inset, Supplementary Fig. 4)[3]. Strong electron exchange interactions at the LL intersection favor the formation of the simplest pseudospin QHF, in which disorder or finite temperature produces a domain wall (DW) separating the two domains with different pseudospin polarizations[4,5]. Charge transport across the DW is assigned to account for an emerging peak (so-

called spike, marked by circle in Supplementary Fig. 4) within the persistent conductivity minima that is the signature of QHF. The line shape and position of the spike are found to be the same for both samples, while a large spike amplitude for the Hall bar is caused by edge transport across the DW (see main text).

The temperature dependence of conductivity minima in the QHF region is shown in Supplementary Fig. 7, which is well fitted with variable-range hopping theory[6]. We expect that hopping transport between charged topological defects (Skyrmion-like) trapped in DW[4] dominates the dissipative process as the network of DWs forms a percolation cluster through the entire 2D plane[7]. It is shown that the derived characteristic temperature T_0 ($\propto 1/\xi$, ξ is the localization length) in the two samples is similar, suggesting that the domain/DW morphology is independent of sample geometries. This helps us to demonstrate the role of edge states in dynamic nuclear polarization (DNP) by a comparative resistively detected NMR (RDNMR) study.

Supplementary Note 2: Direct current (DC) and AC RDNMR measurements

The temporal evolution of σ_{xx} after a large current is applied to polarize the nuclei around the QHF spike is shown in Supplementary Fig. 6a. The RDNMR measurement is performed when σ_{xx} becomes saturated ($\sigma_{xx}^{\mathrm{sat}}$) (see Methods) and the RF frequency (f) dependence of $\Delta\sigma_{xx}/\sigma_{xx}^{\mathrm{sat}}$ represents the RDNMR spectrum. It is shown in Supplementary Fig. 6b that the critical current density needed for the DC RDNMR measurement of the Corbino disk is much smaller than that of the Hall bar. This suggests that the current flowing along the lowest edge state of the Hall bar (corresponding to the pseudospin-up [(n, σ) = (0,↑)] LL, inset of Supplementary Fig. 4) is much larger than the current flowing across the DW that contributes to DNP. In the comparative RDNMR study, we set the operating current to approach the maximum amplitude of $\Delta\sigma_{xx}/\sigma_{xx}^{\mathrm{sat}}$ in each sample (I = 0.6 μA for the Corbino disk and I = 1 μA for the Hall bar).

The dependence of DC RDNMR signals of both samples on different cool-downs is shown in

Supplementary Fig. 2, where the signal amplitude of the Hall bar is found to depend on different cool-downs while that of the Corbino disk does not. We assign the edge transport in the Hall bar to account for this difference: a random impurity potential in different cool-downs will modify the local chemical levels near DW that leads to a change in the transmission probability for the electrons to pass along or across the DW[8] and thus in $\Delta\sigma_{xx}/\sigma_{xx}^{\mathrm{sat}}$. Furthermore, it is found that the direction of current flow and the orientation of magnetic field change the signal amplitude of the Hall bar but do not affect that of the Corbino disk (Fig. 2b and Supplementary Fig. 1). This difference is also induced by the edge transport. It is shown in Fig. 3a,b,g,h that the direction of current flow and the orientation of magnetic field determine the preferred path of edge current. Different paths may lead to a difference in the transmission probability for the electrons to pass across the DW in bulk, which accounts for the difference in the amplitude of bulk signals.

The RDNMR signal obtained from the AC measurement is quite different from the DC counterpart for both Corbino disk and Hall bar. It is seen from Supplementary Fig. 3 that there is no AC signal in the Corbino disk, while a weak AC signal is present at B = 12 T but absent at B = -12 T in the Hall bar. These results provide support for the edge mode of the Hall bar as discussed below. The AC current with opposite flow directions is believed to suppress the bulk DNP of the Hall bar (see main text). However, the direction of edge current flow depending on the orientation of B cannot be changed by the AC current, making the polarized nuclei with opposite spins distribute along the length rather than the width of DW. The spatial overlap between the two species varies with the DW length that may be modified by external factors (magnetic field, cool-down, etc.), which accounts for diverse RDNMR responses (one example is given in Supplementary Fig. 3).

Supplementary References

1. McEuen P. L., Szafer A., Richter C. A., Alphenaar B. W., Jain J. K., Stone A. D., Wheeler R. G. & Sacks R. N. New resistivity for high-mobility quantum Hall conductors. *Phys. Rev. Lett.* **64**, 2062-2065 (1990).
2. Ramvall P., Carlsson N., Omling P., Samuelson L., Seifert W., Wang Q., Ishibashi K. & Aoyagi Y. Quantum transport in high mobility modulation doped $Ga_{0.25}In_{0.75}As$/InP quantum wells. *J. Appl. Phys.* **84**, 2112-2122 (1998).
3. Liu H. W., Yang K. F., Mishima T. D., Santos M. B. & Hirayama Y. Dynamic nuclear polarization and nuclear magnetic resonance in the simplest pseudospin quantum Hall ferromagnet. *Phys. Rev. B* **82**, 241304(R) (2010).
4. Brey L. & Tejedor C. Spins, charges, and currents at domain walls in a quantum Hall Ising ferromagnet. *Phys. Rev. B* **66**, 041308(R) (2002).
5. Jungwirth T. & MacDonald A. H. Resistance spikes and domain wall loops in Ising quantum Hall ferromagnets. *Phys. Rev. Lett.* **87**, 216801 (2001).
6. Shklovskii B. & Efros A. *Electronic properties of doped semiconductors* (Springer-Verlag, Berlin, 1984).
7. Fal'ko V. I. & Iordanskii S. V. Topological defects and Goldstone excitations in domain walls between ferromagnetic quantum Hall liquids. *Phys. Rev. Lett.* **82**, 402-405 (1999).
8. Mitra, A. & Girvin, S. M. Electron/nuclear spin domain walls in quantum Hall systems. *Phys. Rev. B* **67**, 245311 (2003).
